# Supplementary figures and images for: Research on building extraction from remote sensing imagery using efficient lightweight residual network
Source: PeerJ Comput Sci. 2024 May 2;10:e2006. doi: 10.7717/peerj-cs.2006 (PMC11157594; doi:10.7717/peerj-cs.2006)

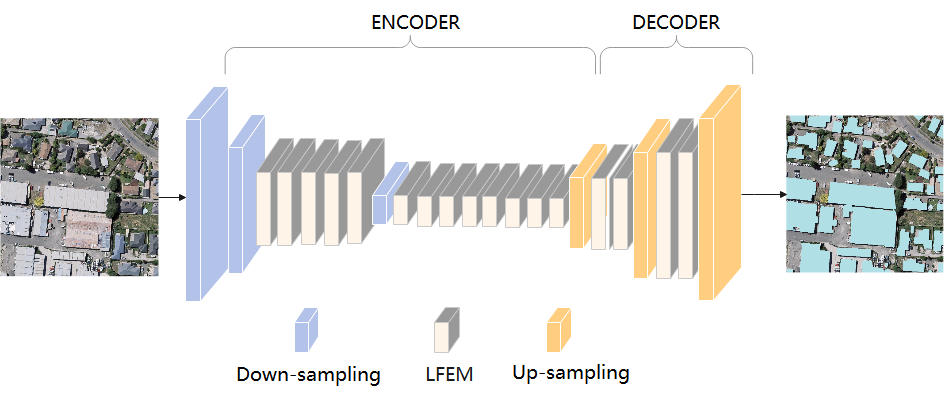

Supplement: Supplemental Information 1 [file peerj-cs-10-2006-s001.zip › Code/ELRNet/Architecture.tif]

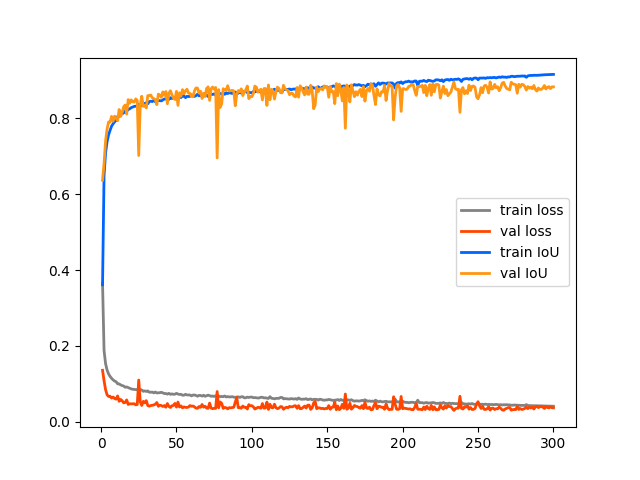

Supplement: Supplemental Information 1 [file peerj-cs-10-2006-s001.zip › Code/ELRNet/ELRNet/loss_and_IoU.png]
